# Allelic Variation and Differential Expression of the mSIN3A Histone Deacetylase Complex Gene *Arid4b* Promote Mammary Tumor Growth and Metastasis

**DOI:** 10.1371/journal.pgen.1002735

**Published:** 2012-05-31

**Authors:** Scott F. Winter, Luanne Lukes, Renard C. Walker, Danny R. Welch, Kent W. Hunter

**Affiliations:** 1 Laboratory of Cancer Biology and Genetics, National Cancer Institute, National Institutes of Health, Bethesda, Maryland, United States of America; 2 Department of Cancer Biology, University of Kansas Medical Center, Kansas City, Kansas, United States of America; University of Washington, United States of America

## Abstract

Accumulating evidence suggests that breast cancer metastatic progression is modified by germline polymorphism, although specific modifier genes have remained largely undefined. In the current study, we employ the MMTV-PyMT transgenic mouse model and the AKXD panel of recombinant inbred mice to identify AT–rich interactive domain 4B (*Arid4b*; NM_194262) as a breast cancer progression modifier gene. Ectopic expression of *Arid4b* promoted primary tumor growth *in vivo* as well as increased migration and invasion *in vitro*, and the phenotype was associated with polymorphisms identified between the AKR/J and DBA/2J alleles as predicted by our genetic analyses. Stable shRNA–mediated knockdown of *Arid4b* caused a significant reduction in pulmonary metastases, validating a role for *Arid4b* as a metastasis modifier gene. ARID4B physically interacts with the breast cancer metastasis suppressor BRMS1, and we detected differential binding of the *Arid4b* alleles to histone deacetylase complex members mSIN3A and mSDS3, suggesting that the mechanism of *Arid4b* action likely involves interactions with chromatin modifying complexes. Downregulation of the conserved *Tpx2* gene network, which is comprised of many factors regulating cell cycle and mitotic spindle biology, was observed concomitant with loss of metastatic efficiency in *Arid4b* knockdown cells. Consistent with our genetic analysis and *in vivo* experiments in our mouse model system, *ARID4B* expression was also an independent predictor of distant metastasis-free survival in breast cancer patients with ER+ tumors. These studies support a causative role of *ARID4B* in metastatic progression of breast cancer.

## Author Summary

A person's individual genetic background influences not only the likelihood of developing breast cancer, but also the likelihood of that cancer becoming metastatic. The identification of metastasis susceptibility genes using human samples is rendered impractical by the high degree of genetic diversity among people. Our laboratory's strategy is to cross genetically defined inbred mouse strains to recapitulate a degree of genetic diversity that is more readily studied. By breeding these panels of inbred mouse crosses to a mouse model of breast cancer, we can identify regions of the genome that correlate with observed phenotypic variation including metastatic density and then identify individual candidate genes. This manuscript describes the identification of *Arid4b* as a candidate gene of interest and the experiments we performed to validate its role in metastasis. High expression of *Arid4b* enhances cell migration and invasion and, conversely, knockdown of *Arid4b* inhibits metastasis of breast tumor cells to the lungs. The mouse gene and human *ARID4B* are highly conserved, and among women with ER+ tumors *ARID4B* expression level is predictive of which patients will progress to develop metastatic disease.

## Introduction

Breast cancer remains the most commonly diagnosed malignancy among women in the United States [1]. Because the vast majority of breast cancer related mortality is attributable to disseminated metastatic disease, a clear need exists to identify factors that modulate breast cancer metastatic progression. In addition to acquired somatic mutations, there is accumulating evidence that the genetic background on which a tumor arises can influence disease progression [2]. Identifying and characterizing metastasis susceptibility genes would provide additional insights into the mechanisms associated with tumor dissemination and growth, leading not only to better understanding of this complex process but also ultimately to new targets and strategies for clinical intervention.

Due to the complex interactions between inherited factors and somatic mutations in metastatic progression, as well as the genetic complexity of human populations, identification of inherited susceptibility genes directly in human populations is difficult. To circumvent this our laboratory has chosen to apply a systems genetics approach on a mouse model of metastatic luminal breast cancer, the FVB/N-TgN(MMTV-PyMT)^634Mul^ (MMTV-PyMT) transgenic model. The MMTV-PyMT transgenic mouse model, which expresses the polyoma virus middle T antigen under the control of the mouse mammary tumor virus promoter, rapidly develops tumors in approximately 100% of female mammary glands and >85% of these animals develop pulmonary metastases by 14 weeks of age. When the MMTV-PyMT model is bred onto a variety of different mouse strains, the F_1_ progeny display broad and strain-dependent heterogeneity in primary tumor latency, primary tumor growth rate and lung metastatic density [2].

Two strains, the highly metastatic AKR/J and poorly metastatic DBA/2J, were found to have a 20-fold difference in their metastatic capacity but no significant difference in any other measured tumor phenotype. These strains were also the progenitor strains for the AKXD recombinant inbred panel of mice, which consists of more than 20 substrains that are composites of the original parental strains AKR/J and DBA/2J. The MMTV-PyMT model was therefore bred to 18 different AKXD strains, the F_1_ mice were phenotyped with respect to primary tumor latency and burden and lung metastatic density, and the phenotypes were compared to haplotype maps of the AKXD strains to determine quantitative trait loci (QTLs) associated with mammary tumor progression [3]. Subsequently, RNA was also harvested from F_1_ tumors and gene expression analysis was performed to define individual genes whose expression correlated with progression [4].

In this study we have utilized these resources to identify *Arid4b* as a novel candidate metastasis susceptibility gene. Although the precise molecular functions of ARID4B are unknown, it has been shown to associate with the SIN3A histone deacetylase (HDAC) complex [5]. As predicted by the genetic linkage and gene expression data, higher expression of *Arid4b* is associated with more rapid tumor growth in animal models, as well as increased tumor cell motility and invasion. These effects are associated with differential binding of the AKR and DBA alleles of ARID4B to HDAC complex members mSIN3A and mSDS3. ARID4B was also found to bind the mSIN3A-associated breast cancer metastasis suppressor protein BRMS1. Stable shRNA-mediated knockdown of *Arid4b* significantly inhibited the pulmonary metastatic efficiency of orthotopic mammary tumors without inhibiting primary tumor growth. Consistent with impaired metastasis in the *Arid4b* knockdown lines was decreased expression of a recently described metastasis-predictive gene network [6]. High expression of *ARID4B* was associated with an approximately 2-fold increased risk of metastatic progression in human breast cancer patients who were lymph node negative at diagnosis. Taken together these results demonstrate a causal role for *Arid4b* in tumor growth and metastatic progression and suggest that mechanisms of action involve modification of epigenetic state via the mSIN3A complex and regulation of the conserved *Tpx2* gene network.

## Results

### Identification of *Arid4b* as a potential tumor progression gene

**Figure 1:**
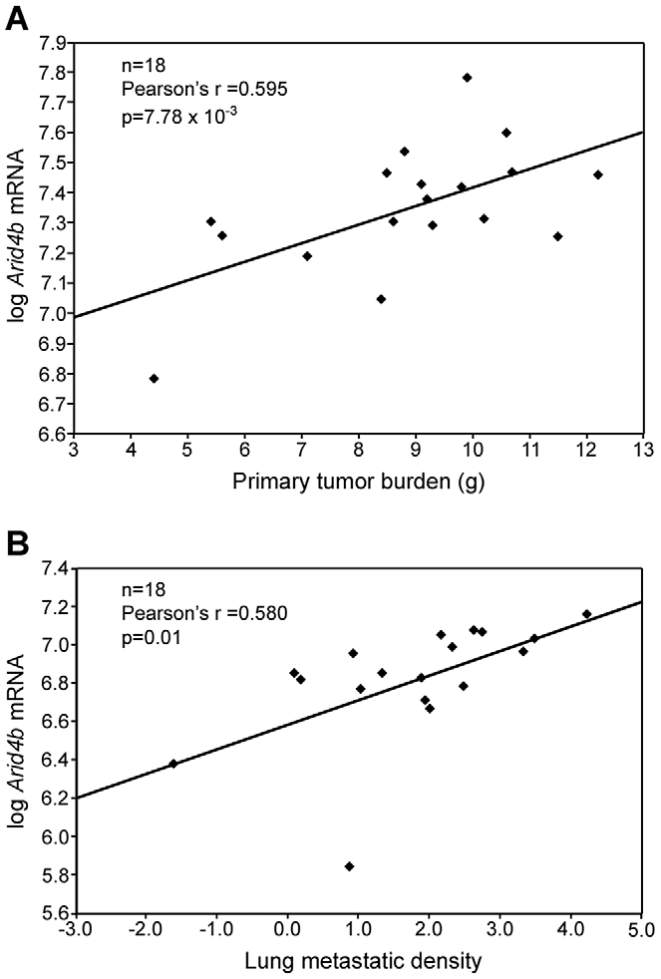
*Arid4b* expression is correlated with tumor growth and metastasis. Gene expression microarray data from 18 of the AKXD recombinant inbred strains shows expression of *Arid4b* is positively correlated with tumor burden (A) and metastatic density (B). The formula used to calculate metastatic density was ln[(metastases/um^2^)×10^8^].

Previously a cross between the highly metastatic PyMT model and the AKXD recombinant inbred (RI) panel was performed to map QTLs associated with inherited predisposition to developing pulmonary metastasis [3]. In addition to metastasis susceptibility loci on chromosomes 6 and 19, linkage analysis revealed a potential peak on proximal chromosome 13 (Figure S1). In a subsequent study, gene expression analysis was also carried out on these samples to examine the effect of varying metastatic genotypes on tumor transcriptional patterns [7]. To discover potential candidate genes that may affect metastatic predisposition, correlation analysis was performed using GeneNetwork [8] to identify genes whose differential expression was highly associated with metastasis. Upon integrating the data from these two studies we found that of the top ten genes most significantly associated with metastasis in our expression correlation analysis, two also mapped to potential QTLs: *Ttc9c* and *Arid4b*. The potential role of *Ttc9c* was investigated and no significant differences were detected with respect to orthotopic tumor growth or metastasis of 6DT1 mouse mammary carcinoma cells stably expressing *Ttc9c* compared to vector control cells (data not shown). Similarly, we detected no significant effects on tumor growth or metastasis when *Ttc9c* BAC transgenic mice were bred to the MMTV-PyMT model (data not shown). The most likely explanation for why *Ttc9c* did not pass our validation experiments is that its initial identification in our screens was a false positive owing to its close physical proximity on chromosome 19 to the metastasis modifier gene *Sipa1* [9]. Our current studies have therefore focused on *Arid4b*, which maps within the chromosome 13 locus and whose mRNA expression was positively associated with metastatic disease and tumor growth (Figure 1), suggesting a possible causative role as a progression modifier.

### *Arid4b* is both differentially expressed and polymorphic between AKR and DBA

**Figure 2:**
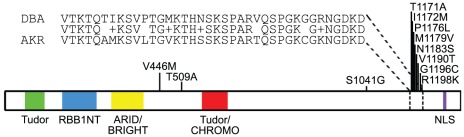
*Arid4b* germline amino acid sequence variants. Substitutions in the AKR allele are shown relative to a consensus sequence that is identical between DBA, FVB, and C57Bl/6. The+symbol indicates conserved substitutions.

To validate the potential differences in *Arid4b* expression between strains, microarray data from AKR and DBA normal tissues were examined [4]. Consistent with the AKXD RI results, *Arid4b* expression was 2.3-fold higher in thymus (p = 9.32×10^−5^, FDR = 0.0004) and 2.5-fold higher in bone marrow (p = 1.28×10^−5^, FDR = 0.0005) of DBA mice compared to AKR, suggesting that constitutional polymorphisms can influence *Arid4b* expression levels in normal tissues. Sequence analysis was also performed to both validate SNPs in the public database as well as identify potential new variants between the AKR and DBA alleles of *Arid4b*. Complete exon sequencing revealed that the DBA allele matched the consensus C57BL/6 sequence. Analysis of the AKR allele revealed numerous silent SNPs as well as polymorphisms encoding eleven amino acid substitutions, as shown in Figure 2. Interestingly, eight of these eleven polymorphisms are located in exon 22 and their encoded substitutions are densely clustered towards the C-terminal end between amino acids 1171 and 1198. These results are consistent with the possibility that inherited variation of *Arid4b* may contribute to tumor progression.

### *Arid4b* expression promotes primary tumor growth

Analysis of the data revealed that increased *Arid4b* expression and increased metastatic susceptibility were associated with the DBA rather than the AKR genotype at the chromosome 13 QTL. This result suggests that the DBA allele at this locus promoted metastatic progression relative to the AKR allele, and a series of *in vitro* and *in vivo* assays were performed to test this hypothesis. V5-tagged AKR and DBA alleles of *Arid4b* were ectopically expressed in the mouse mammary carcinoma cell line Met-1, which was originally derived from tumors arising in the MMTV-PyMT transgenic model [10]. Because the Met-1 line was derived from an FVB strain background, we also sequenced the FVB allele of *Arid4b* and found it to be identical to the DBA and C57BL/6 alleles. Cell lines were then identified that expressed the epitope tagged constructs at levels that were only two to three-fold higher than endogenous levels as measured by QRT-PCR (Figure S2A), consistent with the approximately two-fold range of *Arid4b* mRNA between high and low metastatic AKXD strains. Furthermore, the ectopically expressed AKR and DBA alleles were detected at approximately equal levels in our stable lines as assessed by western blots (Figure S2B).

**Figure 3:**
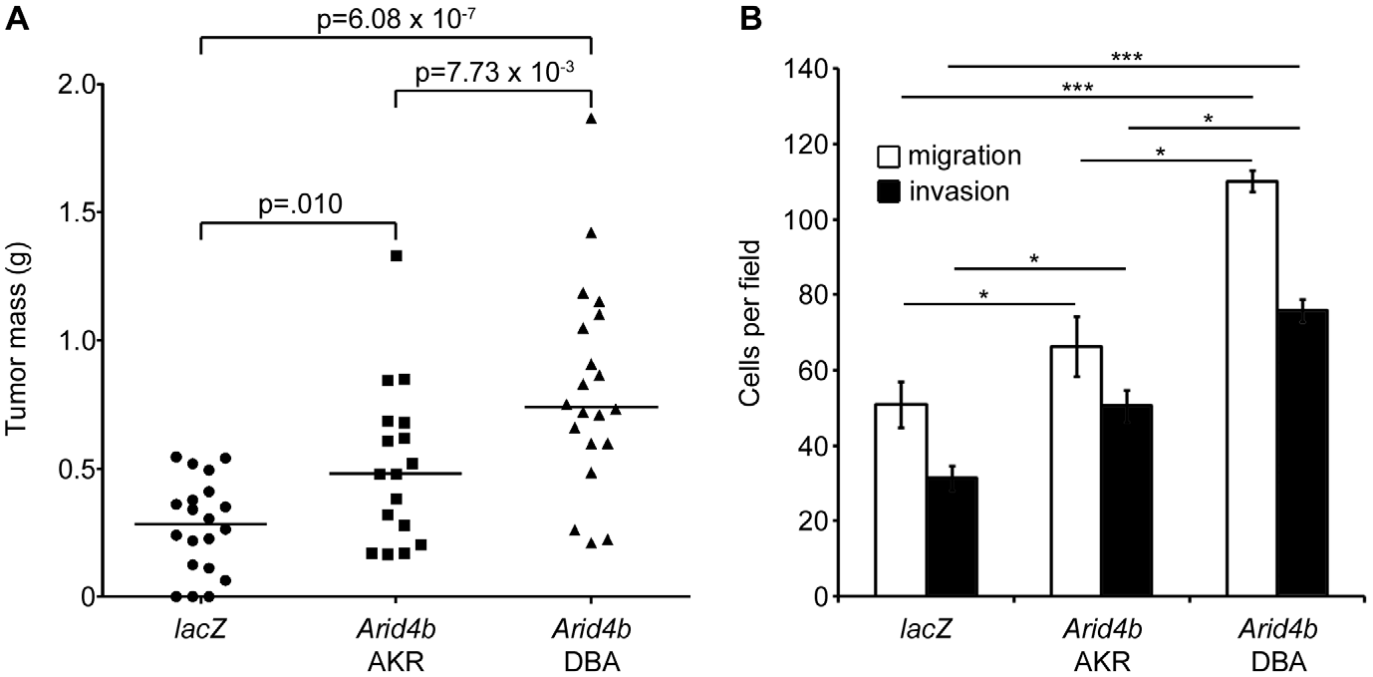
Ectopic expression of *Arid4b* increases orthotopic tumor growth, tumor cell migration, and invasion. Scatter plot bars represent median tumor mass (A) four weeks after orthotopic implantation of 10∧5 cells stably expressing *lacZ* (n = 20), *Arid4b* AKR allele (n = 17) or *Arid4b* DBA allele (n = 20). Migrating and invading cells (B) were counted in five 400× fields per experiment and data are presented as the mean ± standard error of three independent experiments. Statistical significance was determined using Kruskal-Wallis tests followed by Conover-Inman post hoc tests. *, p<0.05; ***, p<0.001.

Orthotopic implantation assays were then performed to examine the role of *Arid4b* expression *in vivo* (Figure 3A). By four weeks post-implantation, cells expressing the DBA allele formed tumors with a 2.6-fold larger mass compared to control cells (741 mg versus 284 mg; p = 6.08×10∧−7). The AKR allele expressing cells formed tumors with a median mass of 480 mg, which was significantly larger than control tumors (p = 0.010) but significantly smaller than the DBA cohort (p = 7.73×10∧−3), consistent with our previous genetic analysis and our *in vitro* studies.

### DBA *Arid4b* promotes increased *in vitro* tumor invasion and migration compared to AKR

*In vitro* assays were performed to address the potential affect of the *Arid4b* polymorphisms on tumor cell behavior. *In vitro* growth assays demonstrated no significant difference in proliferation between cells expressing the DBA or AKR alleles or control cells (data not shown). In contrast, ectopic expression of either allele significantly increased the abilities of Met-1 cells to migrate through a porous membrane and to invade through Matrigel, compared to control cells expressing *lacZ* (Figure 3B). Notably, Met-1 cells stably expressing the DBA allele were significantly more migratory and invasive than those expressing the AKR allele. Since both cell lines express the epitope-tagged construct at approximately the same level, these results suggest a potential functional consequence for the amino acid substitutions present between the two variants in addition to the effects associated with differential expression.

### Knockdown of *Arid4b* inhibits pulmonary metastasis

**Figure 4:**
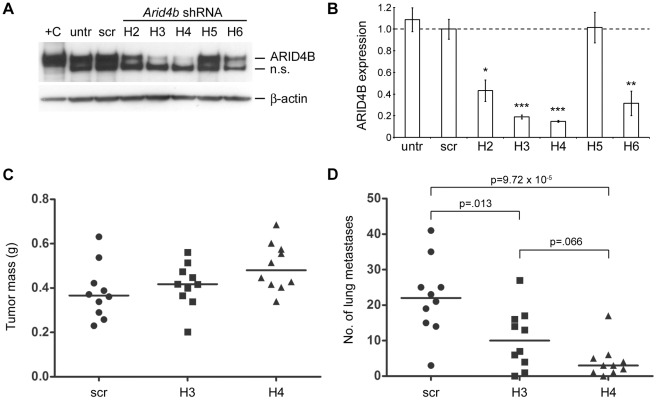
Stable shRNA–mediated knockdown of *Arid4b* inhibits lung metastasis. ARID4B protein levels were analyzed by western blot to determine level of knockdown in cell lines stably expressing different *Arid4b* shRNAs. Experiment was performed in triplicate and a representative blot is shown in panel A. Lysate from 293 cells transiently transfected with *Arid4b* was used as a positive control (+C). Untransduced 6DT1 and scrambled control lines are designated “untr” and “scr” respectively. The lower non-specific (n.s.) band most likely represents ARID4A, based on a nearly identical epitope sequence and consistent with the observed difference in molecular weight. Expression of the non-specific band remained relatively unchanged across the panel of stable lines. Densitometry was performed to quantitate knockdown at the protein level in the *Arid4b* shRNA lines relative to the scrambled control line (B) Data are shown as the mean ± standard error of three independent experiments. Kruskal-Wallis followed by Conover-Inman post hoc tests were used to determine statistical significance versus the scrambled control cell line. *, p<.05; **, p<.01; ***, p<.001. Primary tumors were weighed (C) and lung surface metastases were counted (D) at 3.5 weeks after orthotopic implantation of 10∧5 cells from the scrambled control, H3, and H4 lines into the mammary fat pad of FVB mice (n = 10 per cohort). Horizontal bars represent median values and statistical significance was determined using Kruskal-Wallis tests followed by Conover-Inman correction for multiple comparisons.

Because Met-1 cells are poorly metastatic in our laboratory, and because we were unable to stably overexpress *Arid4b* in several more aggressive mouse breast cancer cell lines, we adopted a knockdown strategy to examine the role of *Arid4b* in lung metastasis *in vivo*. To this end, the highly metastatic 6DT1 cell line [11] was transduced with five lentiviral shRNAs targeting *Arid4b*, or a scrambled control, and knockdown of ARID4B protein was evaluated using western blots (Figure 4A) and densitometry (Figure 4B) to select stable shRNA lines for *in vivo* studies. No significant knockdown was observed using the scrambled control shRNA. Cells stably transduced with *Arid4b* shRNAs designated H3 and H4 expressed 81% and 85% less ARID4B protein, respectively, compared to controls, and were therefore selected for further *in vivo* study.

Following orthotopic implantation of 10∧5 cells into the mammary fat pad we observed only slight differences in median primary tumor mass between the scrambled control, H3, and H4 cohorts, and these data did not achieve statistical significance (p = .070, Kruskal-Wallis; Figure 4C). In contrast, we observed a 2-fold decrease in the median number of macroscopic lung metastases in the H3 cohort (10 vs. 22; p = .013) and a 7-fold decrease in the H4 cohort (3 vs. 22; p = 9.72×10∧−5) compared to controls (Figure 4D). Differences in lung metastasis between the two Arid4b knockdown cohorts were not statistically significant following post hoc testing (p = .066, Conover-Inman). These data demonstrate that ARID4B protein levels are a critical determinant of pulmonary metastatic efficiency in this model system.

### *Arid4b* germline polymorphism modifies binding to mSIN3A

**Figure 5:**
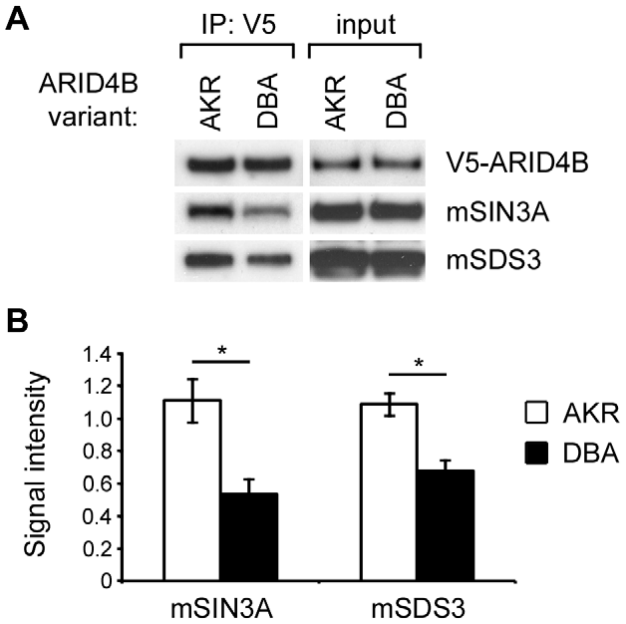
Differential binding of the AKR and DBA variants of ARID4B to the mSIN3A complex. Western blots (A) show equivalent input of V5-ARID4B, mSIN3A, mSDS3, and equivalent pull-down of the two variants using an anti-V5 antibody. Decreased binding of the DBA variant of ARID4B to mSIN3A and mSDS3 was observed relative to the AKR variant. Experiment was performed in triplicate and densitometry analysis (B) revealed a 51% reduction in mSIN3A binding (p = .037) and a 37% reduction in mSDS3 binding (p = .026) for the DBA variant relative to AKR. Statistical significance was determined using paired t-tests. *, p<.05.

Previous studies demonstrated that ARID4B is a member of the mSIN3A HDAC complex and that binding to mSIN3A involves the C-terminal domain of ARID4B [5], where the majority of the amino acid substitutions were found between the AKR and DBA variants (Figure 2). Co-IP analysis was therefore performed to examine a potential effect of the observed amino acid substitutions on ARID4B-mSIN3A binding. For these experiments V5-tagged ARID4B was transiently transfected into HEK293 cells and immunoprecipitated using an anti-V5 antibody. Binding to endogenous mSIN3A and another component of the mSIN3A complex, mSDS3 [12], was evaluated by western blots (Figure 5). Input controls for ARID4B, mSIN3A, and mSDS3 were approximately equal as were the amounts of the two *Arid4b* variants immunoprecipitated; however, a marked decrease in binding to mSIN3A was observed along with diminished mSDS3 association for the DBA variant (Figure 5A). Densitometry analysis revealed that binding of the DBA variant was reduced by 51% and 37% for mSIN3A and mSDS3, respectively, compared to AKR (Figure 5B). These results demonstrate a functional consequence of *Arid4b* polymorphisms and provide insight into one potential molecular mechanism whereby *Arid4b* may modulate breast cancer progression.

### ARID4B binds the breast cancer metastasis suppressor BRMS1

**Figure 6:**
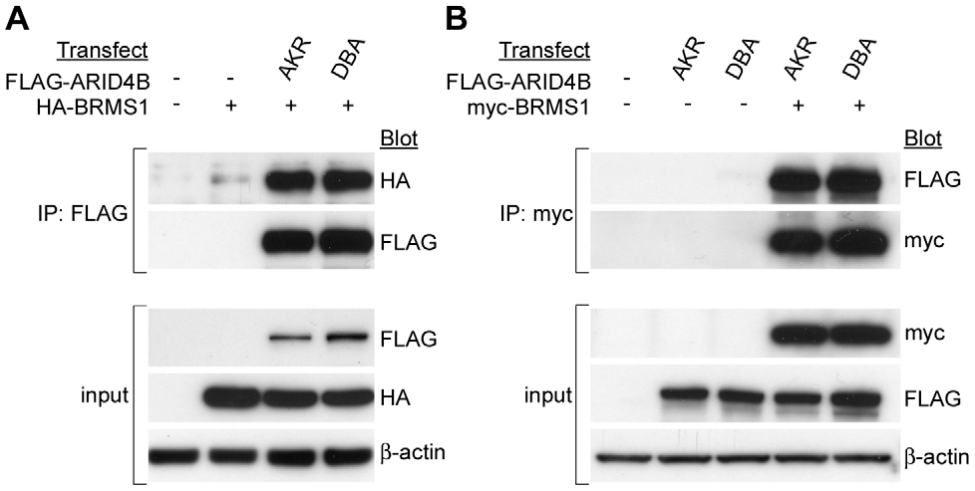
ARID4B binds BRMS1. HA-BRMS1 was coprecipitated with FLAG-ARID4B (A) and FLAG-ARID4B was coprecipitated with myc-BRMS1 (B). No difference in binding to BRMS1 was observed between the AKR and DBA alleles of ARID4B.

Breast cancer metastasis suppressor 1 (BRMS1) belongs to the same family of proteins as mSDS3 and is known to associate with the mSIN3A complex as well as ARID4A [13]. Because ARID4B is also known to bind mSIN3A, mSDS3, and ARID4A [14], we postulated that ARID4B might physically bind BRMS1. Proteomics screens to identify BRMS1 interacting proteins also support this association: in a yeast two-hybrid screen for proteins binding full-length BRMS1, ARID4B was the number one hit identified, and ARID4A and mSDS3 were also detected (unpublished data). In a separate screen, mass spectrometry was performed to identify mSIN3A binding proteins in MCF10A human breast epithelial cells. Peptides representing endogenous ARID4B and BRMS1 were detected, providing further evidence for this interaction and demonstrating that it is not simply an artifact of supraphysiologic expression in transfected cells (Douglas Hurst; personal communication). To validate this interaction we performed co-IPs using lysates from 293 cells transiently transfected with the FLAG-tagged AKR or DBA variants of ARID4B along with either HA- or myc-tagged BRMS1. HA-BRMS1 was readily detected following pull-down of ARID4B using an anti-FLAG antibody (Figure 6A). Likewise, ARID4B was efficiently co-precipitated with myc-BRMS1 (Figure 6B). Unlike the associations with mSIN3A and mSDS3 however, the AKR and DBA variants of ARID4B did not exhibit differential binding to BRMS1. One possible explanation for this observation is that BRMS1 binds to a different region of ARID4B than the polymorphic C-terminal domain that mediates binding to mSIN3A.

### *Arid4b* regulates the metastasis-associated TPX2 gene network

Because little is known about the specific cellular processes regulated by *Arid4b* that might influence the metastatic phenotype, we performed expression microarray analysis on the 6DT1 cell lines stably expressing *Arid4b* shRNAs to identify genes that are differentially expressed as a function of *Arid4b* levels. Based on the western blot densitometry shown in Figure 4A–4B cell lines expressing hairpins H3 and H4 were chosen to represent the *Arid4b* knockdown cohort, and the control cohort consisted of untreated 6DT1 cells and lines expressing the scrambled control shRNA or hairpin H5. We detected 2,048 unique genes whose expression was significantly different (p<0.05, ANOVA) between the two groups and those with the greatest fold change are summarized in Table 1. While the most highly upregulated genes function in pathways with diverse biological roles, it was noted that among the most downregulated genes were multiple factors associated with centromeres (*Cenpi*, *Cenpq*), microtubule and spindle dynamics (*Kif2c*, *Kif4a*, *Sass6*), and cell cycle regulation (*Ccne*, *Cdc25c*). Consistent with this observation were the results of pathway analysis conducted to identify biological functions impacted as a consequence of *Arid4b* knockdown (Table 2). The most differentially regulated processes based on gene ontology were checkpoint control and DNA repair, and processes related to centrosome, centriole, and chromosome dynamics.

**Table 1:** Genes most highly up- or down-regulated following *Arid4b* knockdown.

Gene	Fold Change	p-value
*Sprr1b*	2.931	2.31E−02
*Btg2*	2.761	7.99E−03
*Ctxn1*	2.341	1.93E−02
*Pmm1*	2.274	1.44E−03
*Zmat3*	2.097	8.27E−03
*Gm5617*	2.080	1.88E−02
*Tp53inp1*	2.065	2.32E−02
*Sesn2*	1.998	5.59E−04
*Gramd1a*	1.965	1.30E−02
*Ak1*	1.946	6.52E−03
*Cenpi*	−3.457	9.76E−03
*Kif2c*	−3.403	2.56E−04
*Ccne2*	−3.198	4.00E−03
*Gpr116*	−2.975	2.21E−04
*P4ha3*	−2.937	4.32E−02
*Kif4a*	−2.882	1.41E−02
*Ncapg2*	−2.777	2.49E−02
*Cdc25c*	−2.709	8.10E−03
*Cenpq*	−2.702	1.04E−02
*Sass6*	−2.701	8.76E−04

**Table 2:** Cellular processes regulated by *Arid4b* knockdown.

Biological function	z-score	p-value	# of genes
Checkpoint control	−2.833	1.29E−16	33
DNA repair	−2.660	2.93E−12	58
Cycling of centrosome	−2.914	3.95E−10	21
Formation of centriole	−2.565	8.44E−09	12
Replication of centriole	−2.268	5.77E−08	11
S phase checkpoint control	−2.119	1.58E−07	10
Repair of cells	−2.168	8.06E−07	18
Orientation of chromosomes	−2.386	2.06E−06	11
Transcription	−2.670	2.50E−05	259
Damage of chromosomes	2.157	4.02E−04	9
Cell death	2.172	6.06E−04	28
DNA damage	2.190	2.05E−03	24

Biological processes are listed in order of statistical significance after applying a cutoff based on z-scores with an absolute value greater than 2. Negative z-scores indicate downregulation; positive z-scores indicate upregulation.

**Figure 7:**
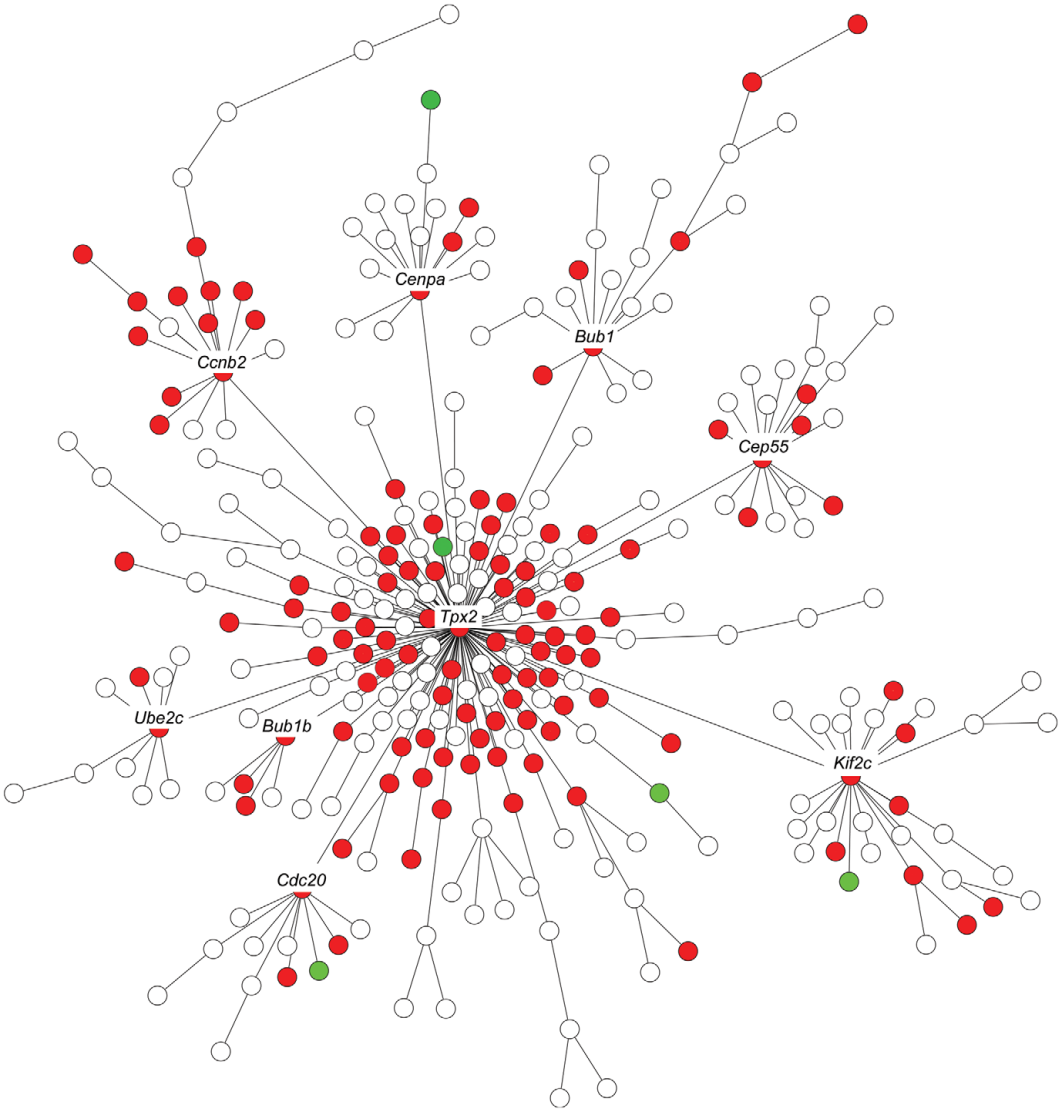
Expression of *Tpx2* network genes in *Arid4b* knockdown cell lines. The nine labeled hub genes were conserved across mouse and human breast cancer data sets. Red indicates statistically significant (p<.05) downregulation; green indicates upregulation.

In examining the microarray data we noticed a striking overlap between genes downregulated in the *Arid4b* knockdown lines and components of the TPX2 gene network. This transcriptional network was recently identified based on expression profiling of three mouse data sets and two human breast cancer data sets [6]. The TPX2 network is tumor cell-autonomous and conserved across species, its activation is predictive of reduced distant metastasis-free survival (DMFS) in ER-positive patients, and the nine common hub genes in the TPX2 signature (*TPX2*, *BUB1*, *UBE2C*, *CDC20*, *CCNB2*, *KIF2C*, *BUB1B*, *CEP55*, *CENPA*) that were conserved across all five data sets consist primarily of genes involved in microtubule and mitotic spindle function. To determine how *Arid4b* levels influence the activation state of the TPX2 network, the fold changes of the 311 TPX2 network genes were examined in the *Arid4b* knockdown lines. Compared to control cell lines, 119 network genes were significantly downregulated (p<0.05) including *Tpx2* itself and the other eight common hub genes, versus only 5 network genes upregulated (Figure 7; high resolution available as Figure S3). The downregulation of this gene network concomitant with the inhibition of metastasis observed in the *Arid4b* knockdown lines provides further support for the role of the TPX2 network in metastatic susceptibility and suggests that a significant portion of this network may be regulated by *Arid4b*.

### High *Arid4b* expression predicts poor clinical outcome

**Figure 8:**
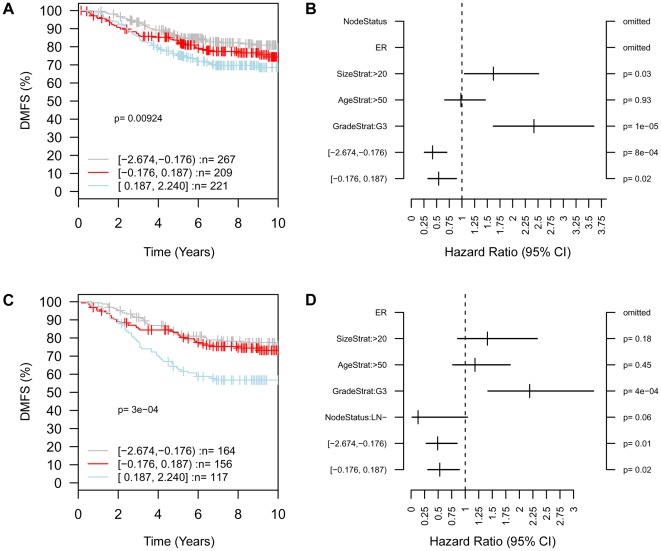
High expression of *ARID4B* is associated with poor clinical outcome. In patients with ER-positive tumors who were node negative at diagnosis (A) distant metastasis-free survival (DMFS) was significantly lower (p = .009) in patients with high expression (blue) compared to middle (red) or low (gray) expression of *ARID4B*, and multivariate analysis of 440 cases (B) was performed to determine metastatic progression hazard ratios of 0.54 and 0.42 for median and low *ARID4B* terciles, respectively, compared to the high *ARID4B* tercile. The association of high *ARID4B* with poor DMFS was also highly significant (p = 3.05×10∧−4) among ER-positive patients in the absence of adjuvant therapy (C) with similar hazard ratios (D) of 0.53 and 0.49 for middle and low *ARID4B* groups compared to high *ARID4B*.

Because *Arid4b* was identified as a candidate gene in part based on differential expression between high and low metastatic strains of mice in the AKXD panel, and because *Arid4b* expression levels were associated with tumor growth and metastasis in mice as well as the activity of the metastasis-associated TPX2 network, we tested whether *ARID4B* expression alone correlated with human patient outcomes. A search of publically available breast cancer microarray data sets using Oncomine (Compendia Bioscience, Ann Arbor, MI) revealed that *ARID4B* expression was 2.3-fold higher in 40 ductal breast carcinoma samples compared to 7 normal breast tissue samples in the Richardson study [15], confirming that high *ARID4B* expression is clinically associated with breast cancer (Figure S4). Analysis of a pooled breast cancer dataset using GOBO (http://co.bmc.lu.se/gobo/) [16] showed that among the subgroup of patients with ER-positive tumors, the cohort with high expression of *ARID4B* had significantly reduced DMFS compared to the low or median *ARID4B* cohorts (Figure 8). Because this association was significant among patients with ER-positive tumors who were lymph node negative at the time of diagnosis (Figure 8A), this finding indicated that *ARID4B* expression level is predictive of patient progression to metastatic disease. As determined by multivariate analysis (Figure 8B), the hazard ratio compared to the high *ARID4B* tercile was 0.54 for middle *ARID4B* (95% C.I. = 0.33–0.89; p = .015) and 0.42 for the low *ARID4B* tercile (95% C.I. = 0.26–0.70; p = 7.51×10∧−4), indicating that patients with tumors expressing high levels of *ARID4B* are approximately twice as likely to develop metastatic disease. The association of *ARID4B* with reduced DMFS was also highly significant among ER-positive patients not receiving adjuvant therapy (Figure 8C–8D), indicating that *ARID4B* expression level plays a significant role in the natural metastatic progression of ER-positive breast cancer in human patients and its relevance is not confined solely to our mouse model systems.

## Discussion

*Arid4b* was identified as a candidate gene of interest through linkage and expression correlation analyses, and the *in vitro* and *in vivo* data presented here provide the first direct evidence of a causal role of *Arid4b* in mammary tumor progression and metastasis. The initial QTL analysis revealed association with *Arid4b* on proximal chromosome 13, and *Arid4b* was among the most highly correlated genes with the most significant *p* values in the subsequent eQTL analysis in the AKXD recombinant inbred panel. It was noted that although AKR/J is the more highly metastatic of the two parental strains, progression was associated with the DBA/2J allele, suggesting that the metastasis promoting influence of *Arid4b* is likely masked by other suppressive factors in a pure DBA/2J background. Although the AKXD recombinant inbred panel lacks the power to detect these epistatic interactions, ongoing experiments using the latest generation of recombinant inbred mice including the Collaborative Cross [17], [18] will enable higher resolution QTL mapping and more robust systems genetics analyses going forward.

Mouse *Arid4b* encodes a protein of 1314 amino acids that shares 89% identity and 95% similarity to the 1312 amino acid human protein. Alternate nomenclature includes breast cancer-associated antigen 1 (BRCAA1), retinoblastoma-binding protein-1-like protein-1 (RBP1L1), and mSIN3A-associated protein of 180 kDa (SAP180). Indeed, there are multiple lines of evidence implicating *Arid4b* in breast cancer. A ten amino acid peptide was found to represent an antigen epitope expressed in 65% of breast cancer specimens and was significantly upregulated in the sera of breast cancer patients compared to healthy donors [19]. ARID4B was also found to associate with the mSIN3A HDAC complex [5], which is in turn known to be bound by the breast cancer associated tumor suppressor ING1 [20], [21], the well-characterized breast cancer metastasis suppressor BRMS1 [13], and the ARID family homolog ARID4A/RBP1 [22], which has also been identified as a breast cancer associated antigen [23].

Ectopic expression of *Arid4b* at a physiologically relevant two- to three-fold increased level resulted in a 3-fold increase in orthotopic tumor mass relative to controls for Met-1 cells expressing the DBA allele, while the AKR allele induced 1.9-fold larger tumors versus control cells. To our knowledge, this is the first direct evidence that *Arid4b* upregulation promotes tumor growth. Although Met-1 orthotopic tumors do not readily metastasize in our experience, transwell assays *in vitro* demonstrated that upregulation of either allele of *Arid4b* increased tumor cell migration and invasion, consistent with a role of *Arid4b* in metastatic progression, and cells expressing the DBA allele were significantly more migratory and invasive than cells expressing the AKR allele. While stable upregulation of *Arid4b* did not induce Met-1 cells to metastasize with any greater frequency, stable knockdown of *Arid4b* in the highly metastatic 6DT1 cell line did cause a dramatic reduction in pulmonary metastases, raising the possibility that *ARID4B* may represent a novel therapeutic target. Taken together, the results of the orthotopic implantation and transwell assays are broadly consistent with our genetic linkage and expression correlation analyses that showed an association of the DBA haplotype on chromosome 13 with metastatic progression in the MMTV-PyMT×AKXD mice, and validate a functional role of *Arid4b* polymorphism in modulating the breast cancer phenotype.

While the molecular mechanisms of *Arid4b* are incompletely understood, an examination of its sequence and conserved domains provides further insight into its potential functions. Arid4b contains a nuclear localization signal (NLS) towards the C-terminus as well as conserved Tudor, RBB1NT, ARID/BRIGHT, and Chromo domains in the N-terminal half of the protein. The ARID domain mediates binding to DNA, although the affinity for AT-rich sequences varies among members of the Arid superfamily [24], [25]. The RBB1NT domain is present in many Rb binding proteins including ARID4A, although it is noteworthy that unlike ARID4A, ARID4B does not contain the LCXCE motif necessary for RB binding [26], and no interaction was observed when we attempted to co-IP ARID4B with RB (data not shown); therefore, the function of the RBB1NT domain of ARID4B remains uncertain. Tudor domains are present in many RNA binding proteins [27] and also bind methylated lysine residues on histone tails [28]. Chromo domain-containing proteins have also been shown to bind methylated lysines and mediate the recruitment of chromatin modifying complexes [29]. Because mSIN3A itself lacks intrinsic DNA binding capability, targeting of mSIN3A-associated HDAC activity depends on interactions with other transcription factors including Mad1 and KLF repressors among others [30]. The presence of putative DNA and histone binding domains in the N-terminal half of ARID4B suggest that its influence on mammary tumor progression involves directing the HDAC activity of mSIN3A complexes to chromatin. This is supported by our observations that the high and low metastatic alleles of ARID4B have a dense cluster of amino acid polymorphisms in the C-terminal domain and bind with different affinities to mSIN3A and mSDS3, though the biochemical significance of this observation remains to be determined. Diminished expression levels or binding affinity of ARID4B may allow mSIN3A to be bound by other proteins with different DNA sequence specificity, perhaps not resulting in a global change in the abundance of any one particular histone mark but rather altering the expression of different subsets of genes.

It is noteworthy that the pro-metastatic ARID4B and the metastasis suppressive BRMS1 bind each other and also to the mSIN3A complex *in vitro*. This observation reinforces the significance of the mSIN3A complex in metastatic progression, and it is tempting to speculate that an HDAC complex may be caught in a molecular tug-of-war between these two metastasis modifier genes. However, the mSIN3A complex is modular in nature and interacts with a great variety of transcriptional regulators [30], [31]. Many different complexes exist, and their precise composition and function within the context of breast cancer are not well understood. Further studies will be necessary to define a role, if any, for the ARID4B-BRMS1 interaction in human disease.

While *ARID4B* expression was not a significant predictor of DMFS across all patients in a meta-analysis of an 1,881 sample data set, statistical significance emerged when patients were stratified based on ER status. The observation that *ARID4B* is predictive of metastatic progression only in ER+ patients is consistent with the identification of *Arid4b* as a candidate gene in the context of the MMTV-PyMT mouse model system, in which tumors arise from a predominantly ER+ luminal epithelial cell population [32]. Loss of ER and PR is detected during progression to late carcinomas, however in a systematic analysis of gene expression profiles these late PyMT tumors clustered most closely with human luminal tumors [33], which are ER+. Also consistent with *ARID4B* promoting metastatic progression of ER+ tumors is our observation that *Arid4b* knockdown caused a significant downregulation of the core components of the *Tpx2* gene network. The *TPX2* signature was tumor cell autonomous and predictive of DMFS only in those patients who were ER+ at diagnosis, and was distinct from a CD53 network that was associated with ER-negative stromal components [6]. Polymorphisms in several other tumor cell autonomous metastasis susceptibility genes identified in our laboratory including *Sipa1*, *Rrp1b*, and *Brd4* are prognostic only in ER+ patients [9], [34]–[37], and stable expression of *Brd4* can also differentially regulate the *Tpx2* network [6]. The association of multiple metastasis susceptibility genes with a transcriptional network comprising many cell cycle and mitotic spindle checkpoint regulatory genes highlights the possibility that these cellular functions are critical determinants of metastatic efficiency. Further experiments are underway in our laboratory to determine whether upregulation of the *TPX2* network is causative in promoting metastasis.

## Materials and Methods

### Identification of *Arid4b* germline polymorphisms

Using genomic DNA from AKR/J and DBA/2J mice as templates, PCR was performed to amplify the protein coding region, exons 2 through 24 (Table S1). PCR Products were subjected to agarose gel electrophoresis, bands isolated using the QIAquick Gel Extraction kit (Qiagen) according to manufacturer's recommendations, and used as templates for sequencing. All sequencing runs were performed by the DNA Sequencing and Gene Expression Core, NCI, Bethesda, MD. Genomic sequences for the AKR and DBA alleles were aligned using pairwise BLAST [38] and non-synonymous polymorphisms verified by manual comparison of chromatograms using Chromas software (Technelysium).

### Expression vectors

V5-tagged AKR and DBA alleles of *Arid4b* were generated using long range PCR with forward primer 5′-AACAAAGGTGCAGGTGAAGC-3′ and reverse primer 5′-CCTGCACTCAACTGACATTCCATTC-3′ to amplify *Arid4b*, and PCR products were cloned into pcDNA3.1/V5-His-TOPO (Invitrogen). FLAG-tagged *Arid4b* vectors were constructed by the Protein Expression Laboratory, SAIC-Frederick, Inc. using Gateway technology (Invitrogen). Briefly, the AKR or DBA allele of *Arid4b* was PCR amplified and cloned into entry vector pDonr-253, then subcloned by Gateway LR recombination into pDest-737 to generate an expression construct with CMV promoter and N-terminal 3xFLAG tag. Full-length BRMS1 was epitope tagged at the N-terminus by PCR with the HA or myc tag sequence incorporated into the forward primer and cloned into pCMV or pcDNA3-hygro (Invitrogen), respectively. Correct sequences of all vectors were confirmed prior to use.

### Cell culture and stable cell lines

Met-1 cells [10] were a gift from Dr. Robert Cardiff (University of California, Davis, CA). 6DT1 cells [11] were a gift from Dr. Lalage Wakefield (NCI, NIH, Bethesda, MD). HEK293 cells were purchased from ATCC (Manassas, VA). Cell lines were maintained in DMEM supplemented with 10% FBS, 2 mM L-glutamine, penicillin and streptomycin. Cells were confirmed to be free of mycoplasma contamination using the MycoAlert detection kit (Lonza).

Met-1 cells seeded onto 10 cm tissue culture plates were co-transfected with 6 µg of the appropriate V5-tagged *Arid4b* construct described above, or pcDNA3.1/V5-His-TOPO/*lacZ* (Invitrogen) as a control vector, plus 600 ng of pSuper.Retro.Puro (Oligoengine) as a selectable marker, using FuGENE 6 transfection reagent. Cells were selected using 1 mg/ml G418 plus 4 µg/ml puromycin and clones derived by limiting dilution. Stable upregulation of *Arid4b* was verified by performing QRT-PCR using forward primer 5′- GGTGAGTGGGAGCTGGTCTA-3′ and reverse primer 5′- ATAAAGGGCCCACTGAAGGT-3′, and western blotting for endogenous and ectopically expressed ARID4B as described below. 6DT1 cells were transduced with one of five different lentiviral shRNAs targeting *Arid4b* (RMM4534-NM_194262, Open Biosystems) or a scrambled control shRNA in the same pLKO.1 vector. Stable cells were selected using 10 µg/ml puromycin and pooled clones were analyzed for *Arid4b* knockdown by western blot.

### Animal studies

Met-1 or 6DT1 stable lines were orthotopically implanted into the fourth mammary fat pad of six week old female NU/J or FVB/NJ mice using 10^5^ cells suspended in 100 µl of PBS per animal. Primary tumors and lungs were harvested 28 days later. All experiments were performed according to the National Cancer Institute Animal Care and Use Committee guidelines.

### Migration and invasion assays

Met-1 cells stably expressing *Arid4b* or *lacZ* were seeded at 75,000 cells per well into invasion chambers coated with Matrigel basement membrane matrix (534480, BD Biosciences) or control chambers lacking Matrigel (354578, BD Biosciences). After 24 hours, cells were fixed in 100% methanol, stained with crystal violet, and mounted onto glass slides using mineral oil. Cells were visualized at 400× magnification and five fields were counted for each of three experiments.

### Co-immunoprecipitations

HEK293 cells were transfected with the V5- or FLAG-tagged AKR or DBA allele of *Arid4b*, with or without HA-BRMS1 or myc-BRMS1 where appropriate, using FuGENE 6 transfection reagent. After 30 hours, cells were harvested in mild IP lysis buffer (25 mM Tris-HCl pH 7.4, 150 mM NaCl, 1 mM EDTA, 1% NP-40, 5% glycerol) supplemented with protease inhibitors (11836170001, Roche) and phosphatase inhibitors (P-5726, Sigma). Protein samples were quantitated using Bradford assays. Gammabind G Sepharose beads (17088501, GE Healthcare) were washed twice in NET buffer (50 mM Tris pH 8.0, 150 mM NaCl, 5 mM EDTA, 1% NP-40, 0.5% BSA, 0.04% sodium azide) supplemented with protease and phosphatase inhibitors, and resuspended to form a 50% bead slurry. Lysates were precleared by adding 40 µl of bead slurry and rotating for 30 minutes at 4°C. Samples were centrifuged at 10,000 rpm for 1 minute at 4°C and pre-cleared supernatant transferred to a fresh tube. Anti-V5, anti-FLAG, or anti-myc tag antibodies was added to a final concentration of 1.0 µg/ml and samples rotated for 1 hour at 4°C, then 50 µl of bead slurry was added and co-IPs performed overnight at 4°C. Beads were then washed four times with NET buffer and resuspended in SDS-PAGE sample buffer.

### Western blots and densitometry

NuPAGE precast gels and buffers (Invitrogen) were used according to manufacturers recommendations and gels were transferred onto Immobilon-P (Millipore). Membranes were blocked in TBS with 0.5% Tween-20 (TBST) plus 5% nonfat dry milk for 1 hour at room temperature then incubated with the primary antibody diluted in blocking buffer overnight at 4°C. The following primary antibodies and concentrations were used: anti-Arid4b (1∶3,000; A302-233A, Bethyl Laboratories), anti-V5 (1∶5,000; 37–7500, Invitrogen), anti-mSIN3A (1∶1,000; sc-994, Santa Cruz), anti-mSDS3 (1∶2,000; A300-235A, Bethyl Labs), anti-β-actin (1∶10,000; ab6276, Abcam), anti-FLAG (1∶3,000; F-3165, Sigma), anti-HA (1∶5,000; 11867423001, Roche), anti-myc tag (1∶2,000; 2276, Cell Signaling). After three washes in TBST, membranes were incubated with one of the following horseradish peroxidase conjugated secondary antibodies diluted in TBST plus 0.5% milk for 1 hour at room temperature: anti-mouse (1∶5,000; NA931V, GE Healthcare), anti-rabbit (1∶10,000; sc-2004, Santa Cruz), anti-goat (1∶10,000; sc-2304, Santa Cruz). Membranes were washed an additional three times in TBST and proteins detected using the Amersham ECL Plus system (RPN2132, GE Healthcare) and Amersham Hyperfilm ECL (28906837, GE Healthcare) according to manufacturer's recommendations. Densitometry data were collected and analyzed using a ChemiDoc-It Imaging System and VisionWorksLS software (UVP).

### Expression profiling and network analysis

Total RNA was isolated from pooled clones of 6DT1 *Arid4b* knockdown cell lines using RNeasy kits (Qiagen) and then arrayed on Affymetrix GeneChip Mouse Gene 1.0 ST arrays by the Microarray Core in the NCI Laboratory of Molecular Technology. Expression data were normalized using Partek Genomics Suite to identify genes whose expression was significantly different (p<.05) between the *Arid4b* normal cohort (untreated, scrambled control, and H5 lines) and the *Arid4b* knockdown cohort (lines H3 and H4). The gene list and expression values were then analyzed using Ingenuity Pathways Analysis (Ingenuity Systems, www.ingenuity.com) to identify differentially regulated signaling pathways and biological functions. Expression of the *Tpx2* transcriptional network was visualized and figure generated using Cytoscape software [39]. Microarray data are available through the Gene Expression Omnibus under accession number GSE35731.

## Supporting Information

Figure S1 Interval mapping for metastatic progression in the AKXD recombinant inbred panel. A potential QTL peak was detected on proximal chromosome 13. Likelihood ratio score (LRS) for correlation with metastasis is shown in blue with the AKR genotype in red and the DBA genotype in green.
(TIF)

Figure S2 Quantitation of *Arid4b* expression in stable cell lines. QRT-PCR data (A) was internally normalized to *Ppib* and fold change expressed relative to Met-1 cells stably expressing *lacZ*. Western blots confirm upregulation of the AKR and DBA alleles at the protein level relative to endogenous expression in *lacZ* controls (B).
(TIF)

Figure S3 High resolution map of *Tpx2* network gene expression in *Arid4b* knockdown cell lines. Red indicates statistically significant (p<.05, ANOVA) downregulation; green indicates upregulation.
(TIF)

Figure S4 *ARID4B* mRNA expression in ductal breast carcinoma versus normal breast tissue. Dots represent minimum and maximum values, whisker bars represent 10^th^ and 90^th^ percentiles, boxes represent 25^th^ to 75^th^ percentiles, and center bars represent median values. Fold change was 2.299 and statistical significance was determined by two-tailed t-test. Figure adapted from Oncomine representation of *ARID4B* expression in the Richardson breast cancer data set [15].
(TIF)

Table S1 *Arid4b* exon amplification and sequencing primers.
(DOC)
