# Supplementary figures and images for: Allelic Variation and Differential Expression of the mSIN3A Histone Deacetylase Complex Gene Arid4b Promote Mammary Tumor Growth and Metastasis
Source: PLoS Genet. 2012 May 31;8(5):e1002735. doi: 10.1371/journal.pgen.1002735 (PMC3364935; doi:10.1371/journal.pgen.1002735)

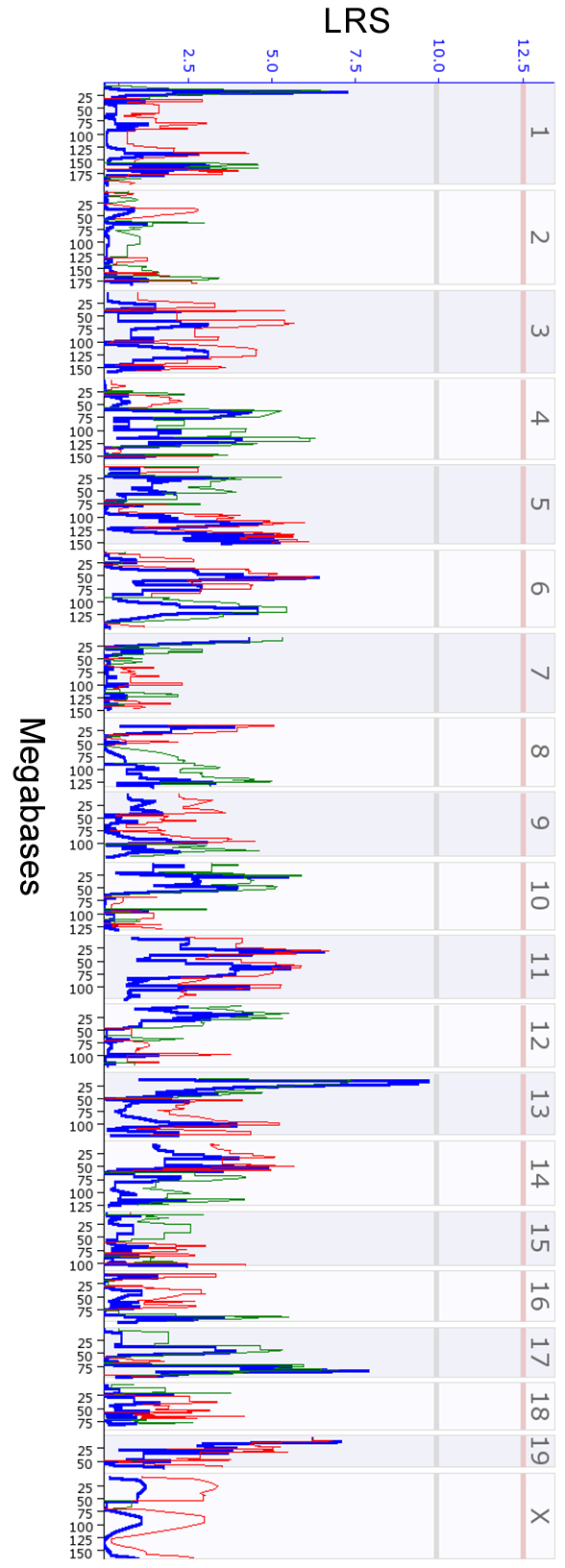

Supplement: Figure S1 — Interval mapping for metastatic progression in the AKXD recombinant inbred panel. A potential QTL peak was detected on proximal chromosome 13. Likelihood ratio score (LRS) for correlation with metastasis is shown in blue with the AKR genotype in red and the DBA genotype in green. (TIF) [file pgen.1002735.s001.tif]

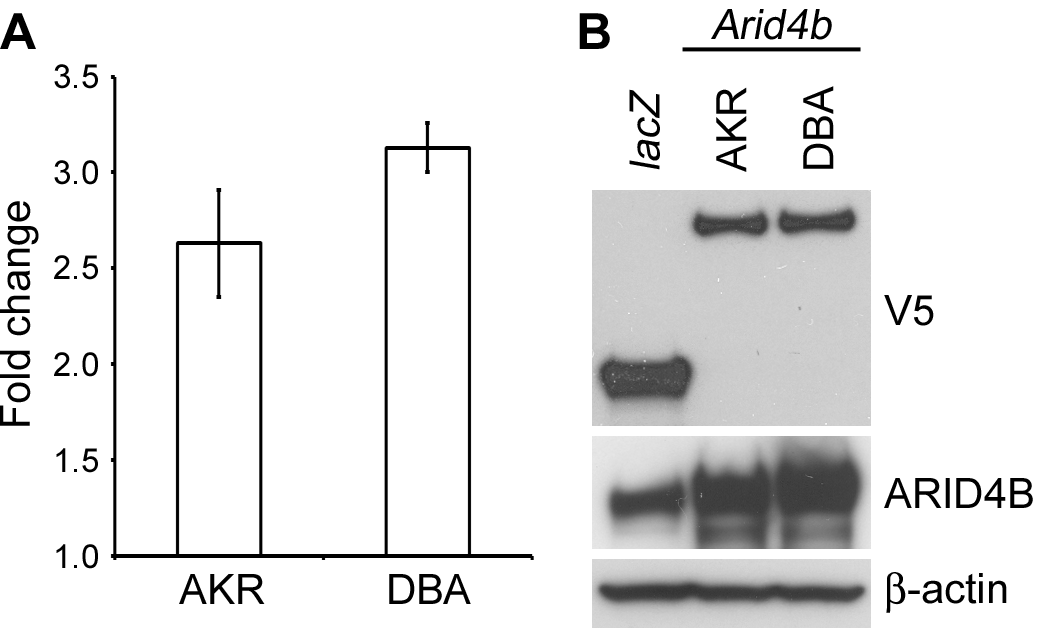

Supplement: Figure S2 — Quantitation of Arid4b expression in stable cell lines. QRT-PCR data (A) was internally normalized to Ppib and fold change expressed relative to Met-1 cells stably expressing lacZ. Western blots confirm upregulation of the AKR and DBA alleles at the protein level relative to endogenous expression in lacZ controls (B). (TIF) [file pgen.1002735.s002.tif]

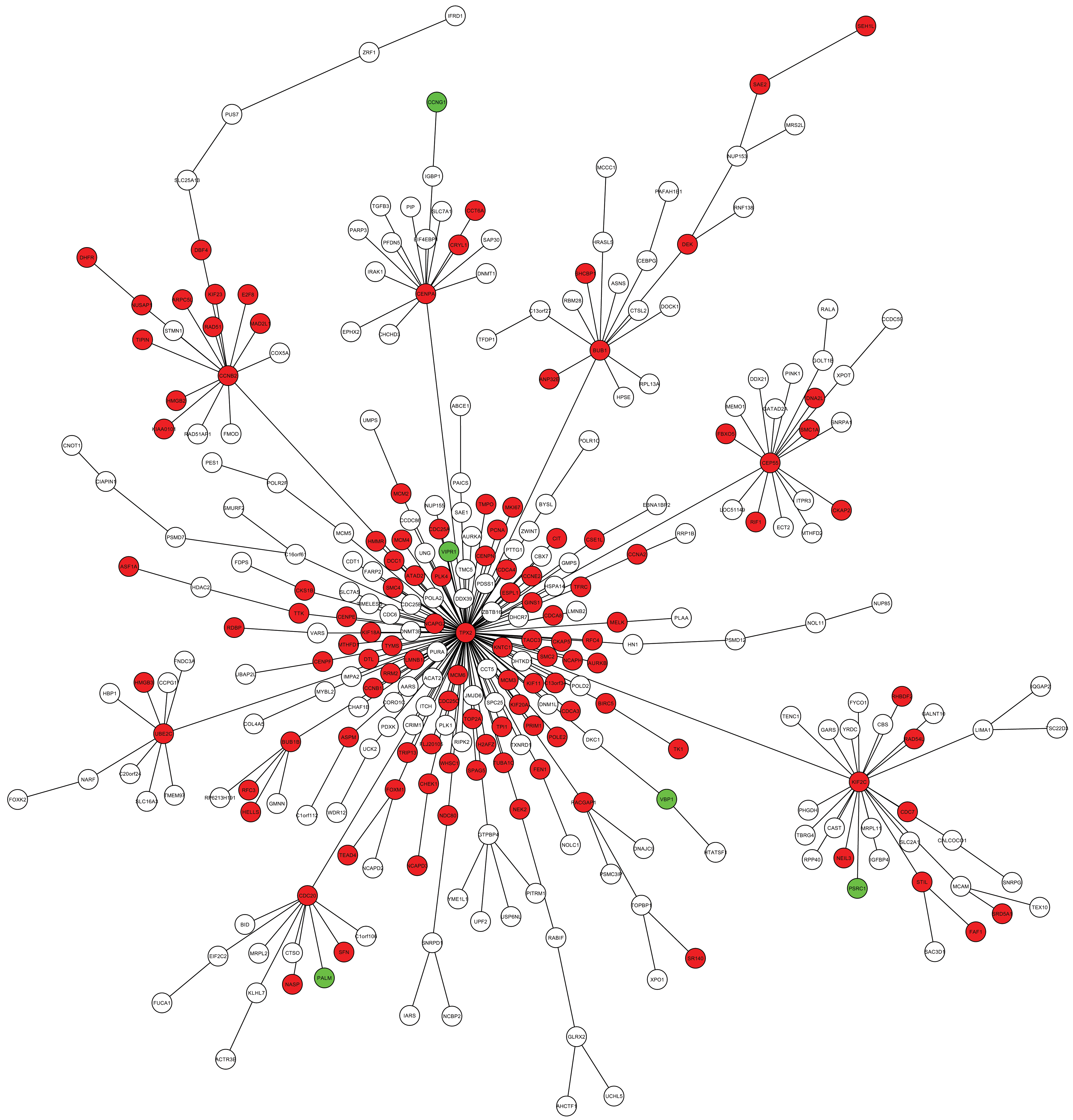

Supplement: Figure S3 — High resolution map of Tpx2 network gene expression in Arid4b knockdown cell lines. Red indicates statistically significant (p<.05, ANOVA) downregulation; green indicates upregulation. (TIF) [file pgen.1002735.s003.tif]

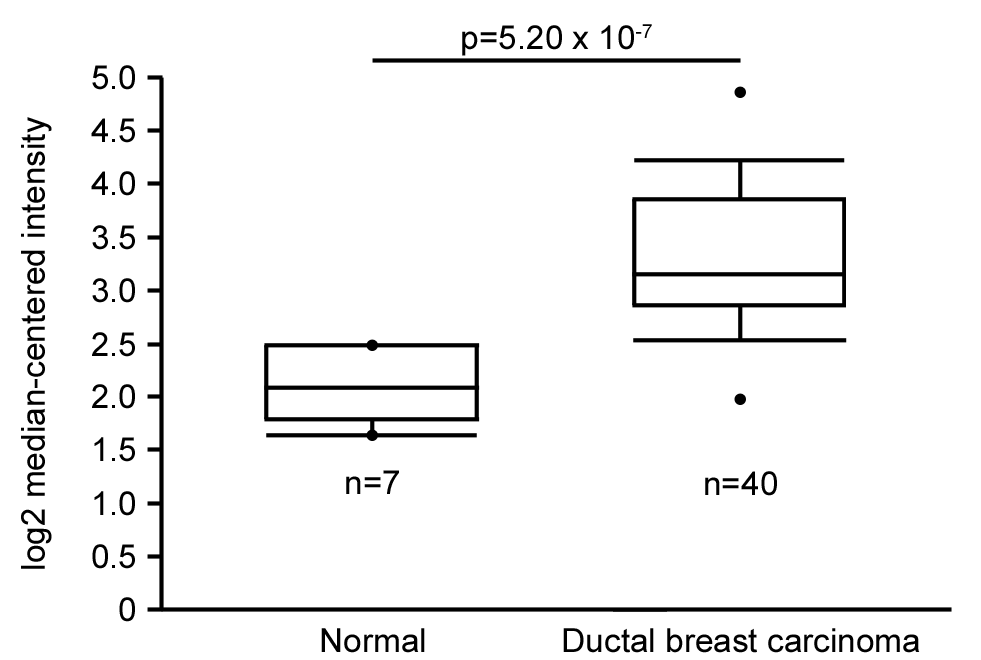

Supplement: Figure S4 — ARID4B mRNA expression in ductal breast carcinoma versus normal breast tissue. Dots represent minimum and maximum values, whisker bars represent 10th and 90th percentiles, boxes represent 25th to 75th percentiles, and center bars represent median values. Fold change was 2.299 and statistical significance was determined by two-tailed t-test. Figure adapted from Oncomine representation of ARID4B expression in the Richardson breast cancer data set [15]. (TIF) [file pgen.1002735.s004.tif]
