# Supplementary material for: Allelic Variation and Differential Expression of the mSIN3A Histone Deacetylase Complex Gene Arid4b Promote Mammary Tumor Growth and Metastasis
Source: PLoS Genet. 2012 May 31;8(5):e1002735. doi: 10.1371/journal.pgen.1002735 (PMC3364935; doi:10.1371/journal.pgen.1002735)
Supplement: Table S1 — Arid4b exon amplification and sequencing primers. (DOC) [file pgen.1002735.s005.doc]

Table S1. *Arid4b* exon amplification and sequencing primers.

| **Primer name** | **Primer sequence (5’ to 3’)** |
| --- | --- |
| Exon 2 forward | CTGACCCGGTGTCTTTGTTT |
| Exon 2 reverse | ACCTGAACAGCTTGGGTGAG |
| Exon 3 forward | TGAAGCCTGATTGTAGGGAAA |
| Exon 3 reverse | GGCAACTTTTGGGATAGCTT |
| Exon 4 forward | TTTGTACATGCATGAGCTGCTA |
| Exon 4 reverse | CACCTGAATGCAAACAGTCAA |
| Exon 5 forward | TGCTTTTGGCTTATTGTCCTC |
| Exon 5 reverse | AGCTAGGACTCCACGTACACA |
| Exon 6 forward | GGCCCAAGAAGGACACTTTT |
| Exon 6 reverse | ACCACAGCCACGGTTACATT |
| Exon 7 forward | AGCCTTCCTTGGATGAAAAA |
| Exon 7 reverse | GACATCAGCAGGAGACCTCAG |
| Exon 8 forward | CCTTGGCCTCCTGACTGTTA |
| Exon 8 reverse | AGCCATCTCTTCAGCTCCAC |
| Exon 9 forward | TGTCCTTGGTTCATCCACAA |
| Exon 9 reverse | CATCCTCTTCGAAGCATTCC |
| Exon 10 forward | TGCATGTTAATGGCCTTGTT |
| Exon 10 reverse | TTTCCTAGGCAAAATGCACA |
| Exon 11 forward | GTACCAGGAGCCTTGCACAC |
| Exon 11 reverse | CTAATCCCCCAAGCACACAT |
| Exon 12 forward | TCAAGAAATGTTGCTTGCATT |
| Exon 12 reverse | TTGGTCTATGAGGTTGGGTTG |
| Exon 13 forward | TAATGGAGCCACGGGTTTAG |
| Exon 13 reverse | AGCACAGCTGCAACACATTC |
| Exon 14 forward | TTGAAAGTTTTTCTTTCCATTTGTC |
| Exon 14 reverse | GGGTCAAAGAACACCCACTG |
| Exon 15 forward | AACCCCACGTACTGTTCAGC |
| Exon 15 reverse | TTTGAGGAACTTTACAAAGGATG |
| Exon 16 forward | GTCACCTCCTGTTGCTGACA |
| Exon 16 reverse | CGACTGTATCCAACCAAAGTG |
| Exon 17 forward | GGAACAATCACAGCCACGAT |
| Exon 17 reverse | TCTGTAATAATCACTAGCTGAAACTCA |
| Exon 18 forward | GAGGGTTTAGGAGGGTTTGG |
| Exon 18 reverse | CCATGCCCACACATGTATTT |
| Exon 19 forward | GCTAACATCCAATGCTTTATGTTTT |
| Exon 19 reverse | TGCCTCAGCTTTCCGAGTAT |
| Exon 20A forward | TGGGAGGTAGGGCATTGTAG |
| Exon 20A reverse | TGGTGTCATCTTTGCTTTGG |
| Exon 20B forward | ACAAAACGTGGGAAAAGACG |
| Exon 20B reverse | CCACTGCTCGGAAATTCTGT |
| Exon 20C forward | AGCTTCTCCACCCCATCCT |
| Exon 20C reverse | CAAGCACACAGCTCAACCAC |

Table S1 cont.

| Exon 21 forward | TGCTGGACTGGATGTGTAGC |
| --- | --- |
| Exon 21 reverse | TAGGTGTGCACCAACTGTCC |
| Exon 22 forward | TTGCTAGGCAAGCATTACACA |
| Exon 22 reverse | GCCATGTCTCTCAGGCTCA |
| Exon 23 forward | GCACCAACACACATGGAAAG |
| Exon 23 reverse | TGAAGCTGACAGCGTAAAGAA |
| Exon 24 forward | TGCAGGGATGGCTCCTATAC |
| Exon 24 reverse | TGTGTGTTTGTGTGCCACTG |
